# Graphene Oxide versus Carbon Nanofibers in Poly(3-hydroxybutyrate-co-3-hydroxyvalerate) Films: Degradation in Simulated Intestinal Environments

**DOI:** 10.3390/polym14020348

**Published:** 2022-01-17

**Authors:** Ariagna L. Rivera-Briso, José Luis Aparicio-Collado, Roser Sabater i Serra, Ángel Serrano-Aroca

**Affiliations:** 1 Biomaterials and Bioengineering Lab, Centro de Investigación Traslacional San Alberto Magno, Universidad Católica de Valencia San Vicente Mártir, 46001 Valencia, Spain; aribri@mail.ucv.es; 2 Centre for Biomaterials and Tissue Engineering, Universitat Politècnica de València, 46022 Valencia, Spain; joapcol@upvnet.upv.es; 3 CIBER-BBN, Biomedical Research Networking Centre in Bioengineering, Biomaterials and Nanomedicine, 46022 Valencia, Spain

**Keywords:** PHBV, films, graphene oxide nanosheets, carbon nanofibers, degradability, acid medium

## Abstract

Poly(3-hydroxybutyrate-co-3-hydroxyvalerate) (PHBV) is a microbial biodegradable polymer with a broad range of promising industrial applications. The effect of incorporation of low amounts (1% *w/w*) of carbon nanomaterials (CBNs) such as 1D carbon nanofibers (CNFs) or 2D graphene oxide (GO) nanosheets into the PHBV polymer matrix affects its degradation properties, as it is reported here for the first time. The study was performed in simulated gut conditions using two different media: an acidic aqueous medium (pH 6) and Gifu anaerobic medium. The results of this study showed that the incorporation of low amounts of filamentous 1D hydrophobic CNFs significantly increased the degradability of the hydrophobic PHBV after 3 months in simulated intestinal conditions as confirmed by weight loss (~20.5% *w/w* in acidic medium) and electron microscopy. We can attribute these results to the fact that the long hydrophobic carbon nanochannels created in the PHBV matrix with the incorporation of the CNFs allowed the degradation medium to penetrate at ultrafast diffusion speed increasing the area exposed to degradation. However, the hydrogen bonds formed between the 2D hydrophilic GO nanosheets and the hydrophobic PHBV polymer chains produced a homogeneous composite structure that exhibits lower degradation (weight loss of ~4.5% *w/w* after three months in acidic aqueous medium). Moreover, the water molecules present in both degradation media can be linked to the hydroxyl (-OH) and carboxyl (-COOH) groups present on the basal planes and at the edges of the GO nanosheets, reducing their degradation potential.

## 1. Introduction

The development of new materials for biomedical applications constitutes a consolidated area of research of the 21st century and much progress have been achieved in the last two decades [1,2,3,4,5,6,7,8,9,10]. In this regard, polyhydroxyalkanoates (PHAs) are a very promising family of biodegradable and renewable lineal bacterial biopolyester polymers [11,12,13,14,15,16,17,18]. In the PHAs’ family, the poly (3-hydroxybutyrate-co-3-hydroxyvalerate) copolymer, also known as poly (3-hydroxybutyric acid-co-3-hydroxyvaleric acid) or poly (hydroxybutyrate-co-hydroxyvalerate) and abbreviated as PHBV or PHBHV, is one of the most promising biopolymers in biomedicine due to its nontoxicity, biodegradability and high biocompatibility with several types of cells and tissues, and currently produced at large-scale [19,20,21,22,23,24,25,26,27]. PHBV has biodegradation times much longer than other biocompatible polymers, such as poly (lactic acid) (PLA), poly (glycolic acid) (PGA) and poly (lactic acid-co-glycolic acid) (PLGA) copolymers, and the degradation products are basic constituents of human blood, which cause minimal inflammatory reactions [28,29].

Thus, PHBV has shown potential applicability in implants, biosensors, cardiovascular stents, scaffolds for tissue regeneration, controlled drug delivery, absorbable surgical sutures and medical packaging [30,31,32,33,34,35,36,37]. Nevertheless, PHBV presents several drawbacks, such as fragility, low impact resistance and reduced elongation at break when compared to conventional polymers [38,39]. In addition, its surface presents poor cell adhesion and proliferation capacity [38,40] and it does not have antibacterial properties [41]. In this regard, several enhancement strategies have been developed in order to improve its properties for potential advanced applications [42]. Thus, we have recently demonstrated that the PHBV’s physical and biological properties, such as compression performance, thermal behavior, wettability, cell adhesion and proliferation of canine adipose-derived mesenchymal stem cells, and antibacterial activity against *Staphylococcus aureus*, can be significantly enhanced with the incorporation of a low amount (1% *w*/*w*) of carbon nanomaterials (CBNs), such as graphene oxide (GO) nanosheets or carbon nanofibers (CNFs), without producing any cytotoxic effect [43].

The incorporation of CBNs in polymeric matrices such as PLA and polycaprolactone (PCL), also in very low amounts has proved to affect the degradation time of these biodegradable polymers [44,45]. In this study, for the first time, the effect of the incorporation of GO and CNFs on the degradation properties of PHBV films is analyzed in two degradation conditions related to a specific in vivo environment, in particular, the human gut condition. The pH changes from highly acid in the stomach (pH 1.5–2.5) to pH 6 in the duodenum. The pH increases from pH 6 in the small intestine to pH 7.4 in the terminal ileum, drops to 5.7 in the caecum and finally reaches a value of 6.7 in the rectum [46]. Thus, two simulated gut conditions were used here using two different media: an acidic aqueous medium (pH 6) and Gifu anaerobic medium (GAM). GAM reproduces more accurately the gut conditions and is widely used as a simulated intestinal medium for the study of human gut microbes [47,48]. GO nanosheets are 2D hydrophilic CBNs, whilst CNFs are 1D hydrophobic CBNs, both with excellent physical and biological properties [49,50,51,52,53,54,55,56,57]. However, CNFs are filamentous carbon-based materials that present some advantages over GO nanosheets: much higher electrical conductivity and much lower cost [54,58]. GO nanosheets possess oxygen-containing functional groups, hydroxyl (-OH) and carboxyl (-COOH), on the basal planes and at the edges, which render them hydrophilic and soluble in polar solvents [49]. CNFs are broadly proposed to produce conductive composites [58] with great potential in biomedicine due to its high surface area and porosity [59].

We hypothesize here that the incorporation of a low amount of GO nanosheets or CNFs (1% *w*/*w*) into PHBV might affect the degradability of PHBV in human gut conditions reproduced by acid aqueous medium (pH 6) and simulated intestinal medium GAM. Due to the different polarity of CNFs and GO nanosheets, we expect to observe a different effect on the degradation of the polar PHBV hydrophobic biopolyester polymer in this comparative study.

## 2. Materials and Methods

### 2.1. Materials

**Figure 1:**
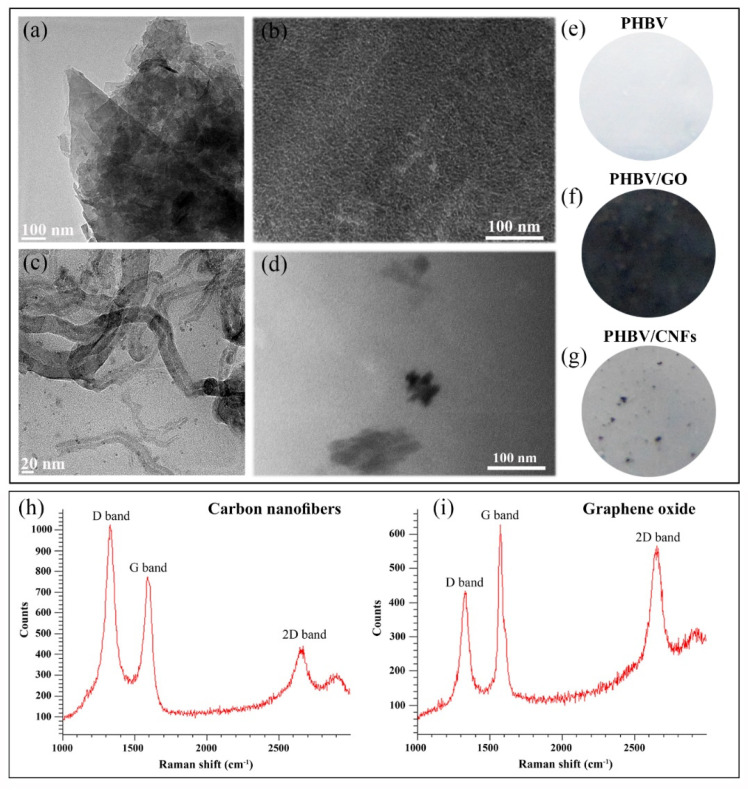
High-resolution transmission electron microscopy images of 2D graphene oxide (GO) nanosheets (**a**) and 1D carbon nanofibers (CNFs) (**c**), transmission electron microscopy images of PHBV/GO (**b**) and PHBV/CNFs (**d**), and photographs of the PHBV (**e**), PHBV/GO (**f**) and PHBV/CNFs (**g**) films. Reprinted with permission from ref. [43]. Copyright 2020 Elsevier. Raman spectra of CNFs (**h**) and GO nanosheets (**i**). Reprinted with permission under a Creative Commons CC BY 4.0 License from ref [60]. Copyright 2020 MDPI.

PHBV, GO (powder, 15–20 sheets, 4–10% edge-oxidized) and dichloromethane (anhydrous, ≥99.8%) were supplied by Sigma-Aldrich (Saint Louis, MO, USA). The CNFs were provided by Graphenano (Yecla, Spain). Gifu anaerobic media (GAM broth) was supplied by Fisher Scientific (Waltham, MA, USA). Sodium azide was purchased from Merck (Darmstadt, Germany). PHBV was previously characterized by nuclear magnetic resonance (NMR) and showed a hydroxylvalerate copolymer mole ratio of 15.23% [43]. This study also showed the morphology of the materials used in the current study (CNFs, GO, neat PHBV, PHBV/GO and PHBV/GO) by electron microscopy (Figure 1a–g).

The Raman spectra of the GO and the CNFs used in this study were also previously measured [60] (Figure 1h,i). The GO can be classified as multilayer GO according to the GRAPHENE Flagship Project of the European Union for the unequivocal classification of these materials [61]. The Raman spectroscopy of the multilayer GO showed a D band intensity/G band intensity ratio (ID/IG ratio) of 0.92, while the CNFs showed a ID/IG ratio of 1.51, which can be attributed to the higher degree of disorder of CNFs [62]. The high-resolution transmission electron microscopy showed the CNFs as one-dimensional hollow filaments with a wide range of diameters (22.7 ± 11.9 nm) and lengths (737.8 ± 522.4 nm). However, the multilayer GO exhibited a morphology of 2D nanosheets with average lateral dimension of 153.8 ± 57.2 nm [60]. The energy-disperse X-ray spectroscopy results showed C/O ratios of 31.3 and 15.4 for the CNFs and GO, respectively. The zeta potential of these GO nanosheets showed a surface charge of −28.94 mV (measured in in water/methanol 98/2) [63]. However, the surface charge of the CNFs used in this study showed a much lower value of −15.4 mV (measured in water at PH = 7) [64]. The dynamic light scattering (DLS) technique showed a particle hydrodynamic size of the GO nanosheets that ranged from 300 to 500 nm, depending on the nanofluid (polyethylene glycol, 1-octadecanothiol, or Triton X-100 surfactant) used for these measurements [65]. However, the DLS hydrodynamic sizes of the 1D nanomaterial showed to be much larger, ranging from 811.2 to 1142 nm, depending also on the nanofluid used (DMEM or water, respectively) [66].

### 2.2. Nanocomposite Film Preparation

Dichloromethane was used to dissolve the PHBV (with and without 1% *w/w* of GO or CNFs). The PHBV-based films were prepared by solvent casting in Petri dishes as previously described [43]. Sonication of CBNs for 30 min was performed in dichloromethane before mixing these dispersions with the PHBV solution. The produced films were finally vacuum-dried at 50 °C for 48 h to constant weight. The neat PHBV sample film and nanocomposite films with GO nanosheets or CNFs are hereafter referred to as PHBV, PHBV/GO and PHBV/CNFs respectively.

### 2.3. Degradation Studies in Acid Medium and Simulated Intestinal Conditions

#### 2.3.1. Degradation Analysis

The quantitative results of this study were based on the weight control of the three types of material films (PHBV, PHBV/GO and PHBV/CNFs) for 3 months in an acid aqueous degradation medium (pH 6). The acid aqueous medium was composed of deionized water with a pH adjusted to 6 with a 10% HCl solution in water. Sodium azide (0.4 mg/mL) was added to the degradation media as fungicide and the pH was monitored during the study to remain constant. Initially, the materials were vacuum dried at 60 °C to determine the dry mass of each material. Samples were introduced into 50 mL falcon tubes containing the degradation medium (20 mL per flask) and placed in the shaking bath with a shaking frequency of 20 cycles per minute at 37 °C. The experimental degradation times were 1, 2 and 3 months. Three replicates of each material per time were analyzed to provide reproducible results. After each selected degradation time, the materials were extracted and dried to constant weight to determine the weight loss and morphological changes.

Gifu anaerobic medium (GAM) broth was additionally used as degradation medium. GAM composition is as follows: 1.0% (*w*/*v*) peptic digest of animal tissue, 0.3% papaic digest of soybean meal, 1.0% protease peptone, 1.35% digested serum, 0.5% yeast extract, 0.22% beef extract, 0.12% liver extract, 0.3% glucose, 0.25% potassium dihydrogen phosphate, 0.3% sodium chloride, 0.5% soluble starch, 0.03% L-cysteine hydrochloride, and 0.03% sodium thioglycollate, final pH 7.3 ± 0.1 [25]. Samples were introduced into 50 mL falcon tubes containing GAM medium (20 mL per flask) and sodium azide (0.4 mg/mL) and placed in the shaking bath with a frequency of 20 cycles/min at 37 °C. The degradation times were 1, 2 and 3 months with three replicates of each material. After each selected degradation time, the samples were extracted and dried to analyze changes in the surface and cross-section morphology.

#### 2.3.2. Electron Microscopy Observation

The qualitative results of the degradation study were evaluated analyzing the morphology (surface and cross-section) of neat PHBV and PHBV with 1% *w/w* of GO and CNFs by high-resolution field emission scanning electron microscope (HRFESEM) using a GeminiSEM 500 microscope (Carl Zeiss, Jena, Germany) at the beginning and at the end of the degradation time (0 and 3 months). The samples were previously coated with a platinum layer by an EM MED020 sputter coater (Leica, Wetzlar, Germany). The cross-section was observed after cryofracture.

### 2.4. Statistical Analysis

Data expressed as mean ± standard deviation (SD). Statistical analysis was performed with the GraphPad Prism 6 (GraphPad Software Inc., San Diego, CA, USA) by one-way ANOVA and subsequent Tukey’s correction. Significant results were assumed at *p*-values < 0.05 (95% confidence).

## 3. Results and Discussion

### Degradation in Simulated Intestinal Conditions

**Figure 2:**
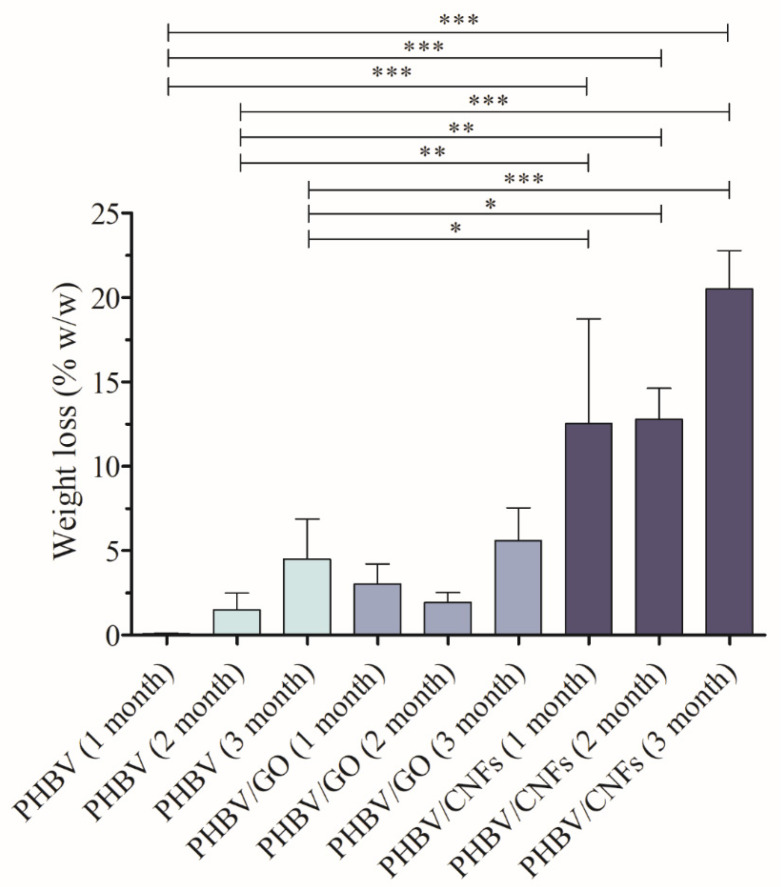
Weight loss of PHBV, PHBV/GO and PHBV/CNFs after degradation in acid aqueous medium (pH = 6) for 1, 2 and 3 months. Data are shown as mean ± standard deviation. Significant differences between samples were calculated by one-way ANOVA with Tukey’s correction. Only the significant differences are indicated in the graph with their level of significance: * *p* > 0.05; ** *p* > 0.01; *** *p* > 0.001.

The results of weight loss after the degradation assay in the acid aqueous medium are shown in Figure 2.

**Figure 3:**
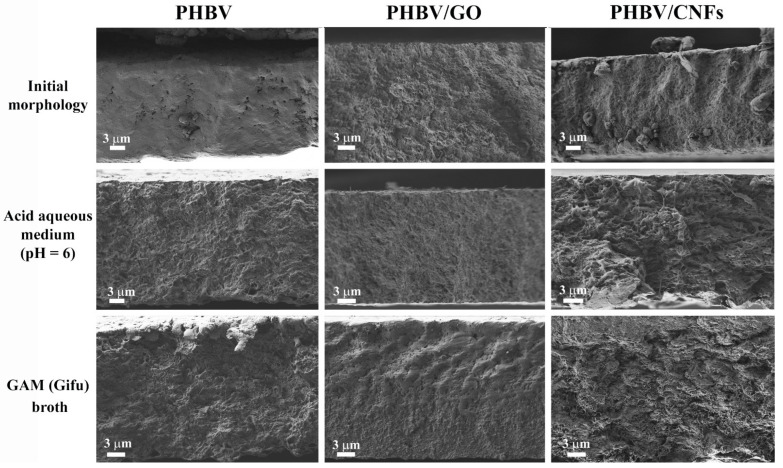
HRFESEM cross-section images of PHBV, PHBV/GO, PHBV/CNFs before (initial morphology) and after degradation in acid aqueous medium (pH = 6) and GAM (Gifu) broth for 3 months.

**Figure 4:**
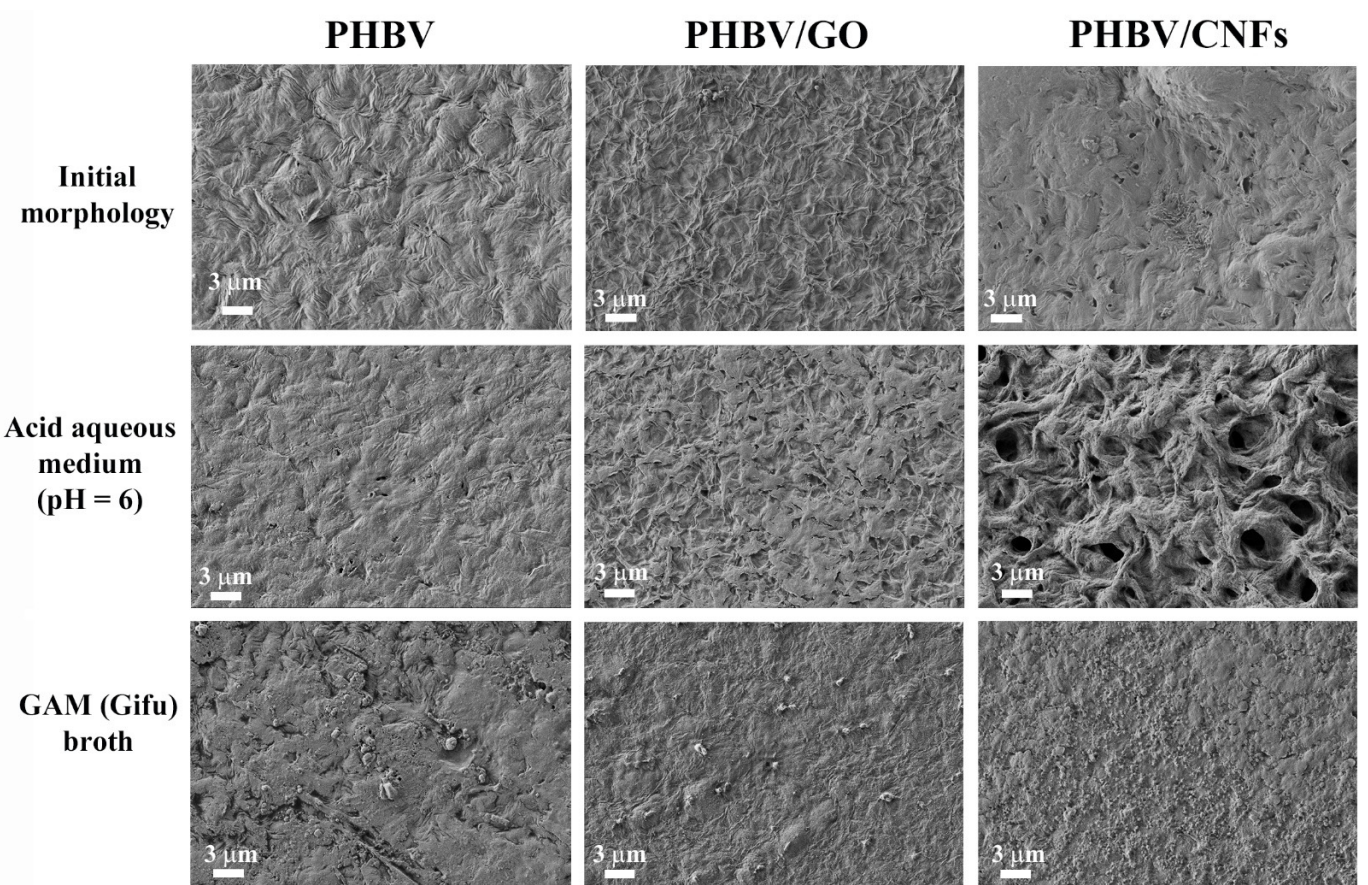
HRFESEM surface images of PHBV, PHBV/GO and PHBV/CNFs before (initial morphology) and after degradation in acid aqueous medium (pH = 6) and GAM (Gifu) broth for 3 months.

The weight loss study shows clearly that the incorporation of 1% *w/w* 1D carbon nanomaterial, CNFs, into the PHBV polymer matrix significantly enhances the degradation properties of the PHBV biopolymer. These results are in good agreement with the HRFESEM cross-section and surface images shown in Figure 3 and Figure 4.

The cross-section images show higher visual degradation in PHBV/CNFs, both in acid aqueous medium (pH = 6) and GAM (Gifu) broth after 3 months (Figure 3). However, the addition of 2D nanomaterial GO (samples PHBV/GO) does not seem to affect substantially the morphology of the samples compared to the images of neat PHBV film. Signs of degradation (changes in the porous structure) can be seen in both neat PHBV and PHBV/GO, although not as evident as those observed in PHBV/CNF samples.

The surface of neat PHBV, shown in Figure 4, shows an irregular morphology, characteristic of semicrystalline polymers [30,63].

The addition of 1% *w/w* of CNFs and GO nanosheets produce slight changes in the surface, although the irregular morphology remains.The surface images of PHBV/CNFs show clearly again the remarkable degradation after 3 months in acid aqueous medium (pH = 6) in good agreement with the weight loss reported in Figure 2. The sample with the 2D nanomaterial (GO nanosheets) also shows signs of surface degradation, although not as noticeable as PHBV/CNF. These signs seem to be related to a first stage of degradation, which has not yet translated into a statistically significant weight loss with respect to pristine PHBV in the analyzed period (Figure 2).

Regarding the surface degradation of PHBV/CNFs films in GAM (Gifu) broth after 3 months, no signs of degradation are observed as evident as in the degradation in the acid aqueous medium. GAM medium contains organic components, such as yeast extract, beef extract and liver extract, among others (see Section 2.3.1) that are deposited on the materials’ surface, preventing the clear visualization of the surface structure. However, as stated previously, the cross-section images showed evident morphological differences of PHBV/CNFs after intestinal degradation compared to PHBV/GO and pure PHBV (Figure 3). Due to the presence of aggregates of organic matter on the surface, observed in all the samples, it was not possible to perform the weight loss analysis, so only the morphological analysis of the samples was carried out after their immersion in GAM medium. It can be noted that the organic layer in pristine PHBV is less dense than in the PHBV/GO and PHBV/CNFs nanocomposites. Several studies have shown that carbon nanomaterials, such as GO, reduced GO (rGO) and CNFs, increase protein adsorption, depending on their concentration or the degree of reduction (in the case of rGO) [67,68]. Proteins spontaneously and quickly adsorb onto carbon nanoparticles, forming the so-called protein corona and covering the surface [69]. The organic components of GAM broth contain a high content of proteins, which are adsorbed to a greater extent on the PHBV/GO and PHBV/CNFs nanocomposites, increasing the density of the surface layer.

Therefore, the results of this study show that the incorporation of a low amount (1% *w/w*) of filamentous 1D hydrophobic CNFs, which are longer nanoparticles and possess lower negative charge than the GO nanosheets [63,64,65,66], significantly increased the degradability of the hydrophobic PHBV after 3 months in simulated intestinal media (both acid aqueous and GAM media). The long filamentous hydrophobic CNFs incorporated into the PHBV polymer matrix creates carbon-based nanochannels through which water (in this case aqueous medium) can penetrate at ultrafast speed according to previous studies [70,71,72], increasing the total area of PHBV chains exposed to degradation. In addition, the hydroxyl groups (-OH) and carboxyl (-COOH) present on the basal planes and at the edges of the GO nanosheets can retain water molecules present in the degradation medium, which reduces their degradation potential. These reactive groups form hydrogen bonds with the PHBV polymer chains producing a homogeneous stable composite structure that prevents the degradation. The weight loss in this nanocomposite presents a similar degradation behavior compared to neat PHBV films, which remains unaltered after the degradation period analyzed in this study.

## 4. Conclusions

PHBV is a microbial renewable biopolymer with great promise in a wide range of biomedical applications, such as controlled drug delivery and tissue engineering, due to its biodegradation and biocompatibility properties. We have demonstrated previously that the physical and biological properties of PHBV can be enhanced with the incorporation of low amounts (1% *w/w*) of 2D or 1D CBNs such as GO and CNFs, respectively. In this study, we show that the degradation time of PHBV in simulated intestinal environments can be reduced with the incorporation of this low amount of CNFs, while the addition of GO nanosheets does not affect the degradability of PHBV in such a significant way. This different degradation behavior can be attributed to the different chemical interactions between hydrophobic PHBV with the hydrophobic CNFs or with the hydrophilic GO. The presence of CNFs in the PHBV polymer produces changes in the structure of the PHBV that accelerate the degradation process, as we have confirmed by the weight loss and electron microscopy analysis.

## Data Availability

Data are contained within the article.
